# Vertexwise Cortical Deviation Mapping (VCDM): A precision approach to cortical thickness assessment in non-affective psychosis

**DOI:** 10.1007/s11682-026-01103-3

**Published:** 2026-02-27

**Authors:** Daniel Mamah, ShingShiun Chen, Fanghong Dong, Michael P. Harms, Mark Curtis, Andrey Anokhin

**Affiliations:** 1 Department of Psychiatry, Washington University School of Medicine, Box 8134, St. Louis, MO 63110 USA; 2 Department of Psychological & Brain Sciences, Washington University School of Medicine, St. Louis, MO USA

**Keywords:** Schizophrenia, Non-affective psychosis, Cortical thickness, Vertexwise, VCDM, MRI

## Abstract

**Background:**

Schizophrenia is associated with structural brain abnormalities, particularly in cortical thickness and gray matter volume. However, despite consistent group-level findings, these measures have had limited clinical utility due to regional variability and the lack of individualized reference frameworks.

**Methods:**

Structural MRI data were obtained from 1,343 individuals across three cohorts: the Human Connectome Project Young Adult (HCP-YA), Early Psychosis (HCP-EP) and a similar mixed cohort (BRAINS). Cortical thickness was assessed in healthy control (CON, n = 1206) and non-affective psychosis (NAP, n = 137) subjects using FreeSurfer and Vertexwise Cortical Deviation Mapping (VCDM) across 298,000 cortical vertices to identify deviations exceeding ± 2 standard deviations from age-adjusted normative values. Group differences and clinical correlations were evaluated using ANOVA and regression analyses. Heritability estimates for deviation metrics were derived from monozygotic and dizygotic twin data.

**Results:**

NAP participants showed significant global cortical thinning compared to controls, with moderate effect sizes (d = 0.53–0.58). Vertex-wise analyses revealed substantially higher percentages of very thin cortical (pTHIN) vertices in NAP (left: d = 1.09; right: d = 1.25), while the percentage of very thick vertices (pTHICK) did not differ by group. Regional analyses of frontal and temporal cortices did not yield improved group differentiation. Clinical symptom severity (e.g., PANSS-positive, SANS scores) predicted higher pTHIN in NAP, while cognitive performance (e.g., working memory, vocabulary) was associated with pTHICK in both groups. Vertex-wise deviation metrics were found to be highly heritable, with heritability estimates of 0.86 (pTHIN) and 0.80 (pTHICK).

**Conclusion:**

VCDM offers a sensitive, individualized method for detecting cortical abnormalities in psychosis, with stronger effect sizes and broader spatial resolution than traditional approaches. It may support more precise diagnosis, prognosis, and treatment monitoring in clinical settings.

## Introduction

Schizophrenia (SCZ) is a disabling psychotic disorder characterized by symptoms that include delusions, hallucinations and disorganized behaviors. With a typical onset usually in late adolescence or early adulthood, SCZ is associated with impaired academic, occupational and/or social functioning (Haas & Sweeney, 1992). Outcomes have been observed to be worse with delayed intervention following illness onset (Byne et al., 2006; Melle et al., 2008), which are linked to progressive brain changes that occur over time (Cahn et al., 2006; Fusar-Poli et al., 2013; Ho et al., 2003; van Haren et al., 2008). Such findings underscore the importance of early intervention in SCZ.

Advancements in brain imaging research hold significant promise for the early identification of individuals with psychotic disorders and may contribute to more precise diagnostic and treatment strategies. Structural brain changes are among the most extensively studied aspects of SCZ and have shown the highest reproducibility, though specific brain regions affected often vary across studies (Haijma et al., 2013; Omlor et al., 2025). Structural findings in SCZ are generally most notable in gray matter and have been linked to illness duration and antipsychotic dose (Haijma et al., 2013). Despite inherent challenges in comparing results across studies due to methodological differences(van Erp et al., 2018), multiple voxel-based morphometry (VBM) meta-analyses have identified gray matter volume abnormalities in individuals with schizophrenia, particularly in the superior temporal gyrus, inferior frontal gyrus, cingulate cortex, thalamus, cerebellum, and insula (Chan et al., 2011; Honea et al., 2005; Qi et al., 2022). A meta-analysis of whole brain studies found decreased cortical thickness in the right inferior frontal gyrus (IFG) and bilateral insula extending to the superior temporal gyrus (Sun et al., 2023). A SCZ meta-analysis conducted by the ENIGMA (Enhancing Neuro Imaging Genetics Through Meta Analysis) Schizophrenia Working Group of an international sample of 4,474 SCZ individuals (mean age: 32.3 years). Compared to controls, those with SCZ had bilateral widespread thinner cortex (Cohen’s *d* > − 0.51) and smaller surface area (*d* = − 0.25), with the largest effect sizes for both in frontal and temporal lobe regions (van Erp et al., 2018). Beyond comparisons of group mean values, those with SCZ have showed greater variability in cortical thickness and surface area within the frontotemporal regions and subcortical volumes (Omlor et al., 2025). Postmortem studies in schizophrenia typically reveal reduced neuropil, smaller neuronal size, and decreased dendritic spine density, leading to increased neuronal packing density, particularly in the prefrontal cortex and hippocampus(Byne et al., 2006; Jaaro-Peled et al., 2010). These microscopic changes underlie macroscopic MRI abnormalities and contribute to disrupted neural networks, cognitive deficits, and psychiatric symptoms.

Despite advances in brain imaging methods, neuroimaging findings have yet to be widely translated into clinical use for psychotic disorders (Dazzan, 2014; McGuire et al., 2015; Scarpazza et al., 2020; Woo et al., 2017). Several factors contribute to this gap, including the non-specificity of imaging findings and disease heterogeneity leading to uncertainties regarding their clinical applicability (Perlis, 2011; Prata et al., 2014; Wardenaar & de Jonge, 2013). Among neuroimaging techniques, structural brain metrics hold strong translational potential, with neuroanatomical measures accounting for up to 40% of the variability in clinical outcomes and can capture aspects of this variability that clinical factors, such as diagnosis, may not account for (Jollans & Whelan, 2016; Scarpazza et al., 2020). Studies have also shown that cortical surface area and thickness are influenced by distinct sets of genes (Panizzon et al., 2009; Winkler et al., 2010). While cortical surface area reductions are often observed in SCZ, most volumetric gray matter changes in SCZ are driven by cortical thinning (Rimol et al., 2012), and cortical thickness appears to be more plastic and influenced by environmental factors, including treatment or substance use (Birnbaum & Weinberger, 2017; van Erp et al., 2018). Previous research in SCZ patients have found regional cortical thickness or volumes to predict treatment response or resistance, often involving frontotemporal regions (Arango et al., 2003; Fan et al., 2023; Goldstein et al., 2002; Mouchlianitis et al., 2016; Szeszko et al., 2012; Zipursky et al., 1998), although translatability is hindered by limited replicability of findings (Mouchlianitis et al., 2016). Several studies have also explored the potential utility of structural brain markers to predict psychosis conversion (Andreou & Borgwardt, 2020). Among individuals at clinical high risk (CHR) from the North American Prodromal Longitudinal Study, accelerated cortical thinning was observed in prefrontal, temporal and parietal regions were associated in those who later converted to psychosis compared to those who did not (Cannon et al., 2015; Collins et al., 2023). These findings suggest that cortical thickness could serve as a valuable biomarker for psychosis-related outcomes.

Traditional methods for measuring cortical thickness have several limitations. Many assess large brain regions, such as Brodmann areas, potentially obscuring localized cortical abnormalities (Furtjes et al., 2023). Voxel-based morphometry (VBM), a widely used neuroimaging technique, enables voxel-wise comparisons to detect subtle regional changes in gray and white matter volume, making it valuable in early-stage or high-risk populations (Ashburner & Friston, 2000). However, VBM does not directly measure cortical thickness or surface area, and its results are highly dependent on preprocessing steps like spatial normalization, segmentation, and smoothing, which can introduce errors (Whitwell, 2009). In addition, partial volume effects can introduce errors in VBM by mixing tissue types within a voxel, potentially masking subtle regional differences in brain volume (Tohka, 2014). Surface-based morphometry (SBM), such as that implemented by the software package FreeSurfer, provides a more anatomically precise alternative by reconstructing the cortical surface and directly measuring cortical thickness as the distance between the pial (outer) and white matter (inner) surfaces (Dale et al., 1999; Fischl et al., 1999). While SBM offers greater accuracy than voxel-based approaches, these methods have not been widely integrated into clinical practice. The absence of a standardized reference framework for evaluating individual cortical thickness data has limited its clinical utility (Tahedl, 2020). Research suggests that establishing region-specific population null distributions and normative modelling is essential for sensitively detecting cortical atrophy in individual patients (Tahedl, 2020; Worker et al., 2023).

This paper introduces Vertexwise Cortical Deviation Mapping (VCDM), a novel whole-brain approach for modeling cortical thickness, and investigates its potential clinical utility in patients with schizophrenia (SCZ) and related psychotic disorders using data from the Human Connectome Project – Early Psychosis (HCP-EP) study. VCDM offers a more detailed assessment of cortical thickness than traditional methods by identifying the proportion of extreme values across nearly 300,000 individual brain vertices. Normative cortical thickness metrics were established using healthy control data, including that from the Human Connectome Project – Young Adult (HCP-YA) and the HCP-EP studies. The following sections detail the application of VCDM to neuroimaging data, with a focus on its potential clinical utility in psychotic disorders.

Our primary hypothesis was that individuals with psychosis would show significantly greater proportions of abnormally thin cortical vertices compared to healthy controls. The overarching aim was to evaluate whether the VCDM approach provides increased sensitivity and clinical relevance over conventional region-based methods for detecting cortical abnormalities in psychosis.

## Methods

### Subjects

Data from three participant groups was used in this study: 1) Human Connectome Project – Young Adult (HCP-YA) participants (n = 1,090), aged 22–37; 2) Human Connectome Project – Early Psychosis (HCP-EP) participants (n = 145), aged 18–34; and 3) additional participants, aged 18–36, recruited as part of an NIH Biobehavioral Research Award for Innovative New Scientists (BRAINS; R01MH104414) that incorporated brain imaging protocols modeled after HCP-YA (n = 108). HCP-YA participants comprised only of healthy control (CON), recruited at Washington University in St. Louis (Van Essen et al., 2012). The HCP-EP cohort comprised of 56 CON participants and 89 non-affective psychosis (NAP) (Jacobs et al., 2024) participants, collected primarily from the Boston, Massachusetts area. The BRAINS cohort comprised of 60 CON and 48 NAP participants, collected from the St. Louis area (Mamah, 2014).

HCP-YA and HCP-EP recruitment and assessment has been previously described (Jacobs et al., 2024; Van Essen et al., 2012). BRAINS participants were diagnosed by a trained clinical coordinator using the Structured Clinical Interview for DSM-IV Axis I Disorders (SCID-IV) (First et al., 2002). CON subjects were required to have no lifetime history of psychotic or mood disorders. Written informed consent was obtained prior to participation, and all study protocols were approved by the Institutional Review Board at the Washington University School of Medicine in St. Louis, MO. All participants were excluded if they: (a) met DSM-IV criteria for substance dependence or severe/moderate abuse during the prior 3 months; (b) had a clinically unstable or severe general medical disorder; or (c) had a history of head injury with documented neurological sequelae or loss of consciousness.

### Image acquisition

HCP-YA image acquisition has been previously described (Glasser et al., 2016). Briefly, subjects were run on a customized Siemens Connectom 3 T scanner, at Washington University in St. Louis, with a 32-channel head coil and completed T1-weighted and T2-weighted structural scans (0.7 mm isotropic). In the HCP-EP subjects, structural T1w MRI image was acquired on a 3 T Siemens Prisma with a 32-channel head coil using a 3D MPRAGE sequence (0.8 mm isotropic voxels, TR/TI = 2400/1000 ms, TE = 2.22 ms, flip angle = 8**°,** FOV = 256 × 240 × 166 mm, matrix size = 320 × 300, 208 sagittal slices, in-plane (iPAT) acceleration factor of 2). T2w volumes were also acquired at the same spatial resolution using the variable-flip-angle turbo-spin-echo 3D SPACE sequence (TR/TE = 3200/563 ms; same FOV, matrix and in-plane acceleration).

A total of 108 BRAINS participants were included in the study. Scans for 60 participants were acquired identically to the HCP-YA protocol, using the Connectom 3 T scanner at Washington University. For the remaining 48 participants, structural T1-weighted MRI data were acquired on a 3 T Siemens Prisma scanner with a 32-channel head coil, using a 3D MPRAGE sequence (0.8-mm isotropic voxels; TR/TI = 2400/1000 ms; TE = 2.22 ms; flip angle = 8°; FOV = 256 × 240 × 166 mm; matrix size = 320 × 300; 208 sagittal slices; in-plane acceleration factor [iPAT] = 2). T2-weighted volumes were also collected at the same spatial resolution using a variable-flip-angle turbo-spin-echo 3D SPACE sequence (TR/TE = 3200/563 ms; same FOV, matrix, and in-plane acceleration).

### Image quality control procedures

All raw NIfTI images were visually inspected prior to HCP pipeline preprocessing (Glasser et al., 2013). T1w and T2w scans were rated on a 4-point scale based on delineation of white–gray matter boundaries, presence of eye-movement artifacts, and banding artifacts; scans rated ≤ 2 were excluded. Surface-level quality control was then performed using the *StructuralQC* scene, which includes each participant’s surfaces, myelin maps, and surface/volume distortion maps (Rosen et al., 2018). Any subject with substantial artifacts (e.g., numerous or large hotspots or black holes in the myelin map) was excluded. Finally, FNIRT nonlinear volume registration outputs were visually examined, and datasets with inaccurate registrations were removed from further analysis. These quality control procedures resulted in the exclusion of one NAP subject from the HCP-EP dataset. No subjects were excluded from the BRAINS dataset.

### Measuring cortical thickness in all subjects

Cortical thickness was measured using FreeSurfer v6.0.0 (Fischl, 2012) with standard preprocessing steps, including intensity normalization, skull stripping, white matter (WM) segmentation, tessellation of the WM boundary, surface smoothing, and automatic topology correction in native space (Fischl & Dale, 2000). A deformable surface algorithm was used to delineate the WM and pial boundaries, from which cortical thickness was calculated. T1w and T2w images were registered within-subject, with T2w scans contributing to bias-field correction, refinement of pial surface placement, and the generation of myelin maps. For group-level analyses, reconstructed surfaces were spatially normalized to the fsaverage template in MNI space, and surface-based smoothing (10 mm full-width at half-maximum [FWHM]) was applied.

### Vertexwise Cortical Deviation Mapping (VCDM)

VCDM was used to identify fine-scale cortical thickness abnormalities. The thickness of 298,261 cortical vertices across the brain were derived from each patient and healthy comparison subjects. For each cortical vertex, we calculated the mean and standard deviation of cortical thickness after correcting for age. To visually identify vertices with outlier values, we color-coded vertices which had cortical thicknesses less than two standard deviations (blue) or greater than two standard deviations (red) from the population mean, each estimated to represent 2.5% of the populating values. A graph was plotted comparing the percentage “thin” cortical voxels (i.e. blue voxels) on the *y*-axis to the percentage of “thick” cortical voxels (i.e. red voxels) on the *x*-axis (pTHIN and pTHICK, respectively).

### Statistical analysis

Statistical analyses were done using SAS 9.4 (SAS Institute Inc., Cary, NC). Group comparisons of structural imaging variables were done using ANOVA, with and without covarying for sex, race and scanner type. Because linear age effects were addressed during preprocessing, a quadratic age term (Age^2^) was also included as a covariate in the ANOVA to account for nonlinear developmental trajectories. To control for multiple comparisons, Bonferroni correction was applied. A linear regression analysis was conducted to examine the relationship between the pTHIN/pTHICK and clinical and cognitive predictors (in psychotic or control groups), depending on what was available for a specific dataset(s). The analysis was performed using stepwise regression with a significance level for entry (SLE) and removal (SLS) both set to 0.05. Effect sizes were estimated using Cohen’s *d*, which quantifies the standardized mean difference between groups. Values of *d* around 0.2, 0.5, and 0.8 were interpreted as small, medium, and large effects, respectively (Cohen, 1988).

For heritability analysis, we analyzed twin pairs from the HCP-YA dataset which was enriched for twins by design. The sample included 337 monozygotic (MZ) twins and 166 dizygotic (DZ) twins. The data were derived from structural MRI scans, and the sample provided a robust basis for investigating the genetic contribution to brain structure variability. Heritability was computed using the standard twin design, which compares the correlation of the trait between MZ and DZ twins. Heritability (h^2^) was calculated using Falconer’s formula:

$${h}^{2}=2\times (r\mathrm{MZ}-r\mathrm{DZ})$$

where *r*MZ is the intrapair correlation between MZ twins, and *r*DZ​ is the intrapair correlation between DZ twins. For each variable (pTHIN and pTHICK), we computed the heritability based on these correlations. Pearson’s correlation coefficients were computed separately for MZ and DZ twin pairs for both pTHIN and pTHICK. Heritability estimates were derived from the difference in correlations between MZ and DZ twins for each variable. These estimates were interpreted as the proportion of variance in the traits explained by genetic factors.

## Results

### Demographic and clinical profile

Table 1 shows demographic and clinical information across the participant groups.

**Table 1:** Shows demographic and clinical information across the participant groups

Characteristic	Control (n = 1,206)	Non-affective Psychosis (n = 137)	t/X^2^	p
Mean age (s.d.)	28.5 (3.9)	23.4 (4.0)	14.5	< 0.0001
Sex, n (%)			28.4	< 0.0001
Female	651 (53.9)	41 (29.9)		
Male	556 (46.1)	96 (70.1		
Ethnicity, n (%)				
			120.6	< 0.0001
Asian	76 (6.3)	6 (4.4)		
Black	181 (15.0)	73 (53.3)		
White	894 (74.1)	51(37.2)		
Mixed	30 (2.5)	1 (0.7)		
Other*	26 (2.2)	6 (4.4)		
Dataset, n (%)			−24.7	< 0.0001
BRAINS	60 (5.0)	48 (35.0)		
Connectom Skyra	33 (2.7)	27 (19.7)		
Prisma	27 (2.2)	21 (15.3)		
HCP-EP	56 (4.6)	89 (65.0)		
HCP-YA	1,090 (90.4)	0		

#### Comparison of cortical thickness across datasets

**Fig. 1:**
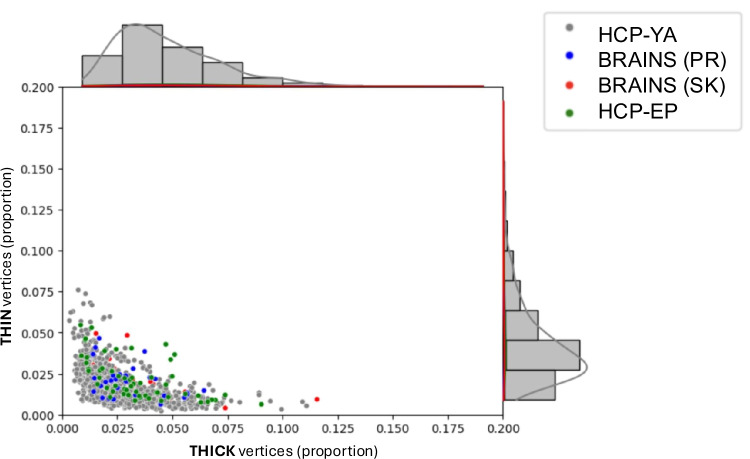
Distribution of VCDM-derived thick and thin cortical vertices across healthy control groups. Each dot shows the proportion of thin and thick mean cortical vertices (from among 298,261 vertices on the cortical surface) of 1,206 healthy participants. Thick cortical vertices refer to the proportion of vertices where cortical thickness is greater than 2 standard deviations from the average of a normative age-corrected sample comprising of four different control participants (n = 1,206). Thin cortical vertices refer to the proportion of vertices where cortical thickness is less than 2 standard deviations from the average of a normative age-corrected sample comprising of four different control participants. VCDM = vertexwise cortical deviation mapping. HCP-YA = Human Connectome Project – Young Adult sample (n = 1,090). BRAINS (PR) = Biobehavioral Research Award for Innovative New Scientists healthy control sample, scanned using a 3 T Siemens Prisma (n = 27). BRAINS (SK) = Biobehavioral Research Award for Innovative New Scientists healthy control sample, scanned using the customized Connectom 3 T Siemens Skyra scanner (n = 33). HCP-EP = Human Connectome Project – Early Psychosis healthy control sample (n = 56)

To verify if structural imaging data is comparable across different datasets using different scanners and minimal protocol differences, we compared quantitative cortical thickness measures from healthy control subjects across datasets. Specifically, we compared average regional cortical thickness (left and right) across the HCP-YA, healthy control HCP-EP, and healthy control BRAINS samples, using ANOVA. Results were highly similar on the left (F = 0.2, p = 0.89) and the right (F = 0.31, p = 0.82) hemispheres. The distributions of pTHIN and pTHICK, defined as the percentage of cortical vertices with thickness values more than 2 s.d. below or above the normative mean, respectively, across healthy control subjects from the different datasets are shown in Fig. 1. Given the high similarity of cortical thickness measures and pTHIN/pTHICK distributions across datasets, cortical thickness values were compared across patient and control groups without within-cohort standardization.

### Global and regional cortical thickness

**Fig. 2:**
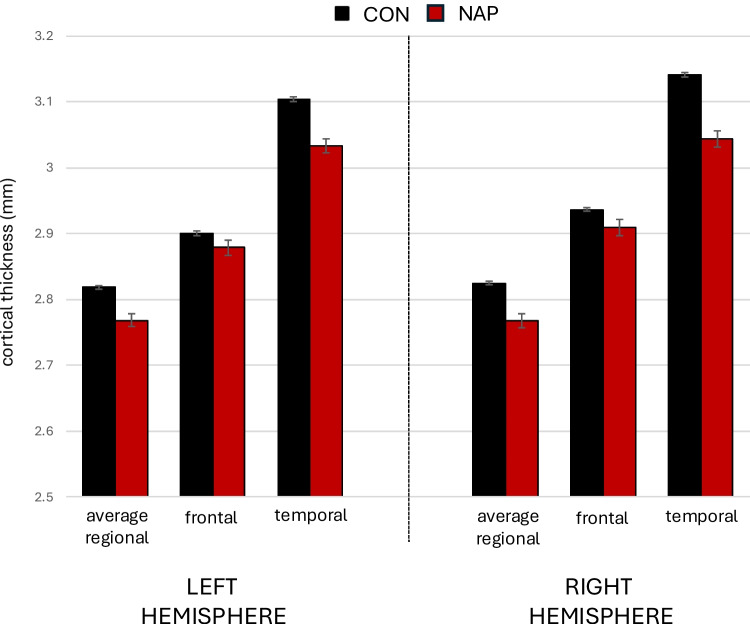
Comparison of regional cortical thicknesses in control and non-affective psychotic participants. Cortical thickness measures were generated from 34 regions per hemisphere using Freesurfer version 6.0.0, based on the standard Desikan-Killiany atlas cortical parcellation. Temporal cortical thickness was based on the average cortical thickness from nine temporal cortex labels (i.e., bank of the superior temporal sulcus, entorhinal, fusiform, inferior temporal, middle temporal, parahippocampal, superior temporal, temporal pole and transverse temporal). Frontal cortical thickness was based on the average cortical thickness from ten frontal cortex labels (i.e., caudal middle frontal, pars opercularis, pars orbitalis, pars triangularis, rostral middle frontal, superior frontal, frontal pole, lateral orbitofrontal, medial orbitofrontal, and precentral). CON = healthy control (n = 1,206). NAP = non-affective psychosis (n = 137)

In CON and NAP groups, we compared the mean global cortical thickness and the mean thicknesses of temporal and frontal cortices, as delineated using FreeSurfer, as depicted in Fig. 2. Mean (s.d.) left-sided cortical thickness was 2.77 (0.11) in NAP and 2.82 (0.09) in CON, and differences were statistically significant (F = 34.8, p < 0.0001). Results were similar when corrected for age (F = 35.4, p < 0.0001) and for age, sex and race (F = 34.8, p < 0.0001). Mean (s.d.) right-sided cortical thickness was 2.77 (0.13) in NAP and 2.82 (0.10) in CON, and differences were statistically significant (F = 42.0, p < 0.0001). Results were similar when corrected for age (F = 42.8, p < 0.0001) and for age, sex and race (F = 42.1, p < 0.0001). The left cortical thinning effect size estimate on the left was d = 0.53, 95% CI [0.68, 0.37] and on the right was d = 0.58, 95% CI [0.73, 0.42], indicating large and meaningful differences between the groups.

Mean (s.d.) left-sided frontal cortical thickness was 2.88 (0.13) in NAP and 2.90 (0.12) in CON, and showed significant group effects (F = 4.6, p = 0.03). Results remained significant when corrected for age (F = 4.7, p = 0.03) and for age, sex and race (F = 4.2, p = 0.04). The left frontal cortical thinning effect size estimate was d = 0.19, 95% CI [0.35, 0.02]. Mean (s.d.) right-sided frontal cortical thickness was 2.91 (0.14) in NAP and 2.94 (0.12) in CON, and showed significant group effects (F = 7.1, p = 0.008). Results were similar when corrected for age (F = 7.3, p = 0.007) and for age, sex and race (F = 6.6, p = 0.01). The right frontal cortical thinning effect size estimate was d = 0.24, 95% CI [0.40, 0.07].

Mean (s.d.) left-sided temporal cortical thickness was 3.03 (0.13) in NAP and 3.10 (0.11) in CON, and showed significant group effects (F = 48.4, p < 0.0001). Results remained significant when corrected for age (F = 49.1, p < 0.0001) and for age, sex and race (F = 24.7, p < 0.0001). The left temporal cortical thinning effect size estimate on the left was d = 0.63, 95% CI [0.77, 0.47]. Mean (s.d.) right-sided frontal cortical thickness was 3.04 (0.15) in NAP and 3.14 (0.11) in CON, and showed significant group effects (F = 91.2, p < 0.0001). Results were similar when corrected for age (F = 92.4, p < 0.0001) and for age, sex and race (F = 93.6, p < 0.0001). The right temporal cortical thinning effect size estimate on the left was d = 0.86, 95% CI [1.00, 0.71].

### Global vertexwise cortical thicknesses compared to the general population

**Fig. 3:**
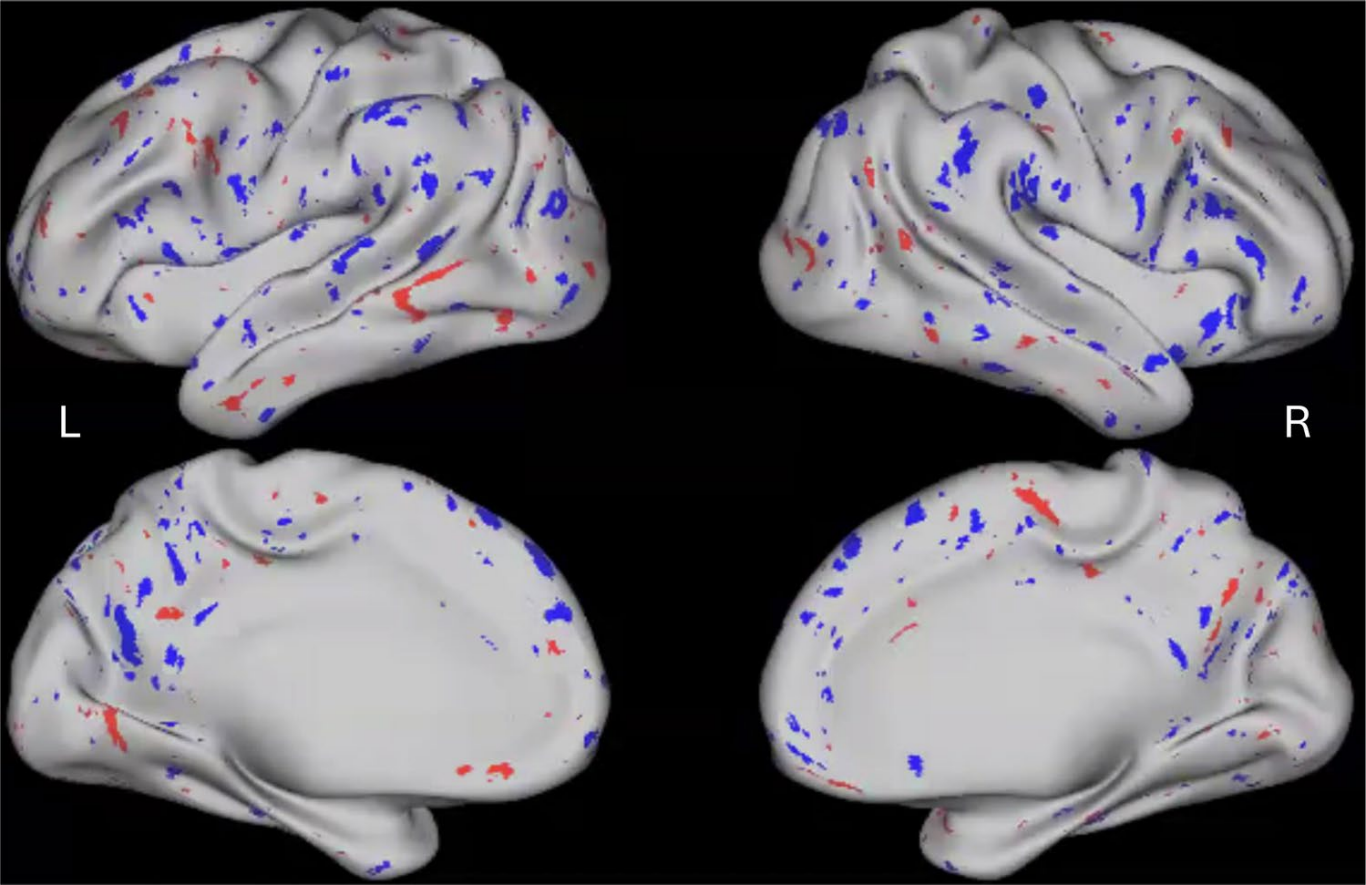
Brain image rendering of VCDM-derived thin and thick cortical vertices in a sample non-affective psychosis participant. Regions with outlier cortical thickness values are depicted among 298,261 cortical vertices on the left (L) and the right (R) hemispheres. Blue vertices indicate regions with cortical thinning (i.e., less than 2 s.d. of the normative population, n = 1,206) and red vertices indicate regions with cortical thinning (i.e., greater than 2 s.d. of the normative population, n = 1,206)

**Fig. 4:**
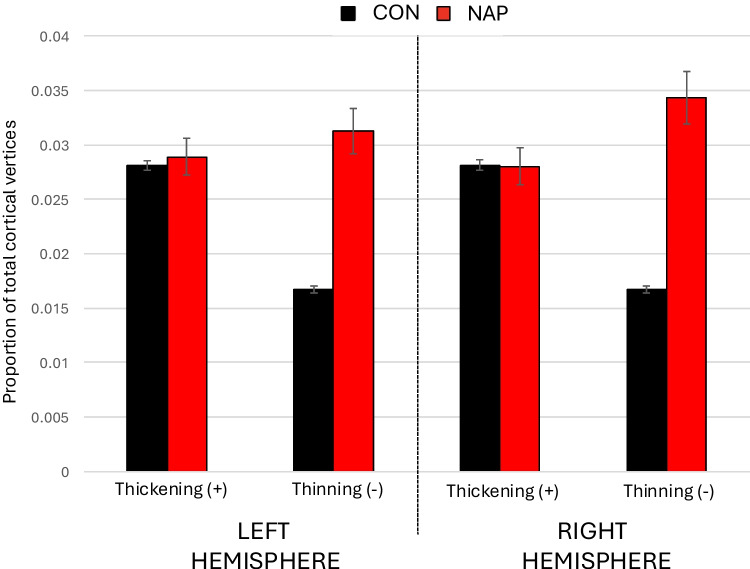
Mean proportion of VCDM-derived thin and thick cortical vertices in control and non-affective psychosis participants. Y-axis shows the mean proportion of 149,130 cortical vertices per hemisphere, showing cortical thinning or cortical thickening. Cortical thickening refers to cortical thickness greater than 2 s.d. of the normative population. Cortical thinning refers to cortical thickness less than 2 s.d. of the normative population. CON = healthy control (n = 1,206). NAP = non-affective psychosis (n = 137)

In each participant, we calculated the average number of outlier thick cortical vertices (i.e., those that were > 2 s.d. of the normative population) and the average number of outlier thin cortical vertices (i.e., those that were < 2 s.d of the normative population). Cortical regions showing thin and thick cortical vertices are depicted in a sample NAP participant in Fig. 3. Mean percentage of thick and thin vertices were compared for the CON and NAP groups in each hemisphere, as shown in Fig. 4.

We found significant group differences in the percentage of very thin cortical vertices. On the left hemisphere, the NAP group showed a mean (s.d.) of 3.13% (0.02) compared to 1.68% (0.01) in controls (F = 145.6, p < 0.0001). On the right, means were 3.43% (NAP) and 1.68% (CON) (F = 192.8, p < 0.0001). Effect sizes indicated large group differences, with d = 1.09 (95% CI [0.88, 1.31]) on the left and d = 1.25 (95% CI [1.03, 1.49]) on the right. Results remained significant after covarying for sex, sex and race, scanner type, or quadratic age effects, with F values ranging from 145.6 to 197.5 (all p < 0.0001).

There were no significant group differences in the average percentages of very thick cortical vertices on the left (CON = 2.81%; NAP = 2.89%; p = 0.6) and on the right (CON = 2.79%; NAP = 2.78%; p = 0.9), with results similar after covarying for sex, scanner type or quadratic age effects.

**Fig. 5:**
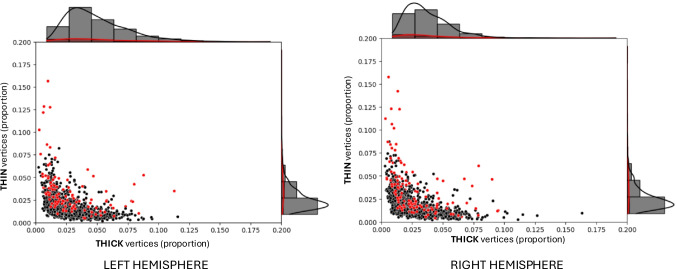
Scatterplots showing the proportion of VCDM-derived thin and thick cortical vertices in control and non-affective disorder participants. The scatterplots show the proportion of thin and thick mean cortical vertices (from among 149,130 vertices on each hemisphere’s cortical surface) of 1,206 healthy participants (black dots) or 137 non-affective psychosis participants (red dots). Thick or thin cortical vertices refer to the proportion of vertices where cortical thickness is greater or less than 2 standard deviations from the average of a normative age-corrected sample (n = 1,206), respectively

Figure 5 shows the distribution of very thin and very thick cortical vertices in each participant. Compared to CON participants, NAP participants tend to have a higher percentage of very thin vertices. Five NAP participants, and no CON participants, had over 10% of very thin cortical vertices. Additionally, the few NAP participants who had over 7% of very thick cortical vertices also had higher percentages (> 4%) of very thin cortical vertices, compared to CON participants with over 7% of very thick cortical vertices.

### Frontal and temporal vertexwise cortical thicknesses compared to the general population

Separately in both the frontal and temporal cortices, we calculated the average number of outlier thick cortical vertices (i.e., those that were > 2 s.d. of the normative population) and the average number of outlier thin cortical vertices (i.e., those that were < 2 s.d of the normative population).

**Fig. 6:**
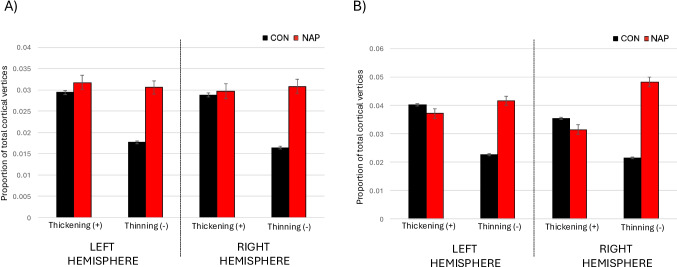
Mean proportion of VCDM-derived thin and thick cortical vertices in frontal and temporal cortical vertices across groups. Y-axis shows the mean proportion of cortical vertices per hemisphere, showing thinning or thickening in the frontal cortex (A) and temporal cortex (B). Cortical thickening refers to cortical thickness greater than 2 s.d. of the normative population. Cortical thinning refers to cortical thickness less than 2 s.d. of the normative population. CON = healthy control (n = 1,206). NAP = non-affective psychosis (n = 137)

In the frontal cortex, we found significant group differences in the percentage of very thin cortical vertices, with the NAP group having an average (s.d.) of 3.06% (0.025) and the CON group having 1.76% (0.016) very thin cortical vertices on the left (F = 68.8; p < 0.0001), and the NAP group having 3.08% (0.029) and the CON group having 1.64% (0.015) very thin cortical vertices on the right (F = 88.2; p < 0.0001) (Fig. 6A). The cortical vertex thinning effect size estimate on the left was d = 0.75, 95% CI [0.96, 0.55] and on the right was d = 0.85, 95% CI [1.06, 0.64]. Differences in the percentages of very thick cortical vertices on the left (CON = 3.17%; NAP = 3.33%; p = 0.2) and on the right (CON = 3.24%; 3.54%; p = 0.6) (Fig. 6A) did not meet statistical significance.

In the temporal cortex, we found significant group differences in the percentage of very thin cortical vertices, with the NAP group having an average (s.d.) of 4.16% (0.036) and the CON group having 2.26% (0.019) very thin cortical vertices on the left (F = 101.8; p < 0.0001), and the NAP group having 4.83% (0.044) and the CON group having 2.15% (0.018) very thin cortical vertices on the right (F = 181.2; p < 0.0001) (Fig. 6B). The cortical vertex thinning effect size estimate on the left was d = 0.91, 95% CI [1.12, 0.71] and on the right was d = 1.21, 95% CI [1.44, 1.13], indicating large and meaningful differences between the groups. Differences in the percentages of very thick cortical vertices on the left (CON = 4.02%; NAP = 3.71%; p = 0.2) and on the right (CON = 3.55%; 3.15%; p = 0.05) (Fig. 6B), but these did not meet statistical significance.

**Fig. 7:**
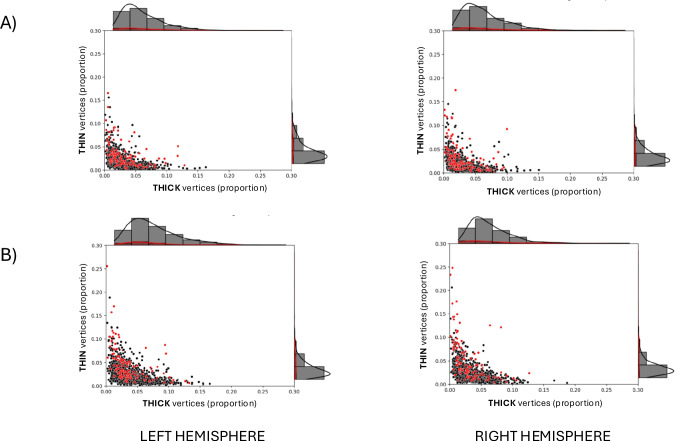
Scatterplots showing the proportion of VCDM-derived thin and thick frontal and temporal cortical vertices across groups. The scatterplots show the mean proportion of thin and thick vertices in each hemisphere among 1,206 healthy participants (black dots) or 137 non-affective psychosis participants (red dots) in the frontal (A) and temporal (B) cortices. Thick or thin cortical vertices refer to the proportion of vertices where cortical thickness is greater or less than 2 standard deviations from the average of a normative age-corrected sample (n = 1,206), respectively

Figure 7 shows the distribution of very thin and very thick frontal and temporal cortical vertices in each participant.

### Clinical and cognitive relationships to vertexwise cortical thickness features

For identify behavioral predictors of the percentages of very thick (pTHICK) and very thin (pTHIN) vertices in HCP-EP psychosis participants, three PANSS domains (positive, negative and disorganized symptoms) and seven cognitive domains (episodic memory, executive functioning, reading/decoding, verbal comprehension, processing speed, working memory and emotion recognition) were included in a stepwise regression model. For pTHIN, the stepwise regression identified the positive PANSS domain as the only significant predictor (R^2^ = 0.0625, F = 5.27, p = 0.02). For pTHICK, the stepwise regression identified working memory as the only significant predictor (R^2^ = 0.0478, F = 3.97, p = 0.0498).

Across healthy control participants (HCP-EP and HCP-YA), there was no significant predictor for pTHIN. For pTHICK, significant predictors included only vocabulary comprehension (R^2^ = 0.0073, F = 8.22, p = 0.0042) and executive functioning (R^2^ = 0.0045, F = 5.11, p = 0.024). When age-adjusted cognitive scores were used for pTHICK, significant predictors included only reading/decoding (R^2^ = 0.0046, F = 5.16, p = 0.023) and emotion recognition (R^2^ = 0.0035, model R^2^ = 0.008, F = 3.9, p = 0.048).

To identify behavioral predictors in BRAINS psychosis participants, three clinical scores (SAPS-positive, SAPS-disorganized and SANS) were included in a stepwise regression model. For pTHIN, the stepwise regression identified SANS score as the only significant predictor (R^2^ = 0.134, F = 7.14, p = 0.01). For pTHICK, the stepwise regression identified SAPS-disorganized as the only significant predictor (R^2^ = 0.0979, F = 4.99, p = 0.03).

As CAINS scores were available for both the HCP-EP and BRAINS samples, we also looked at the relationship of the CAINS with pTHIN across in psychotic subjects across those two datasets. As pTHIN was not normally distributed (Kolmogorov–Smirnov, p < 0.01), Spearman correlations were conducted, which showed a significant relationship (r = 0.237; p = 0.006).

### Heritability of VCDM and regional cortical thickness in HCP-YA samples

Intrapair correlation for pTHIN in MZ twins was 0.70**,** while the intrapair correlation DZ twins was 0.27**,** suggesting strong genetic influences. The heritability estimate for pTHIN was calculated as follows:

$${h}_{\mathrm{p}}^{2}\mathrm{THIN}=2\times \left(r\mathrm{MZ}-r\mathrm{DZ}\right)=2\times \left(0.70-0.27\right)=0.86$$

For pTHICK**,** the correlation between MZ twins was 0.76**,** and between DZ twins**,** it was 0.36**.** The heritability estimate for pTHICK was:

$${h}_{\mathrm{p}}^{2}\mathrm{THIN}=2\times \left(r\mathrm{MZ}-r\mathrm{DZ}\right)=2\times \left(0.76-0.36\right)=0.80$$

**Fig. 8:**
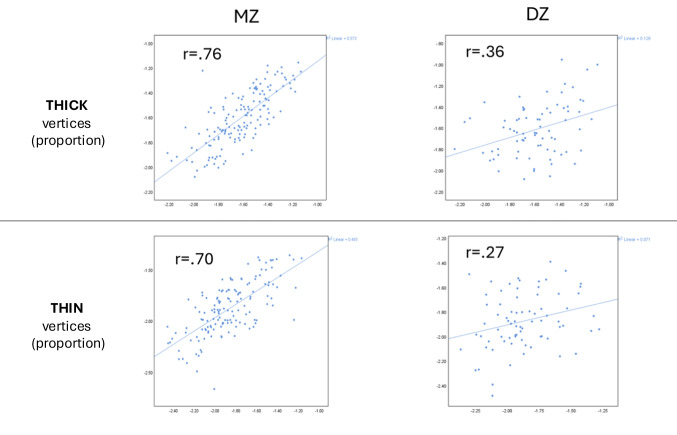
Intrapair twin correlation scatterplots of VCDM-derived thick and thin cortical vertices in Human Connectome Project – Young Adult participants. 337 monozygotic (MZ) twins and 166 dizygotic (DZ) twins from the Human Connectome Project -Young Adult cohort were used. X and Y axes represent phenotype value for the 1 st and 2nd twin in each pair respectively. Each dot represents a twin pair

Scatterplots of the above intrapair correlations are shown in Fig. 8. Both traits showed high heritability, indicating that 86% of variability in pTHIN and 80% of variability in pTHICK is attributable to genetic factors. These were comparable to heritability in cortical thickness measures using regional cortical metrics (Table 2).

**Table 2:** Twin correlations and heritability of cortical thickness metrics in healthy young adults from the Human Connectome Project

Variable	r_MZ_	r_DZ_	Heritability
Mean Cortical Thickness	0.79	0.43	0.72
VCDM Thick Cortical Vertices	0.76	0.36	0.80
VCDM Thin Cortical Vertices	0.70	0.27	0.86

Heritability was computed using the Falconer’s formula: *h*^*2*^ = *2(r*_*MZ*_* – r*_*DZ*_*)*, where r_MZ_ and r_DZ_ are intra-pair correlation coefficients of the neuroimaging phenotypes for monozygotic (MZ) and dizygotic (DZ) twins, respectively.

## Discussion

This study is the first to employ the VCDM approach to assess cortical thickness in individuals with psychosis. Traditional methods of measuring cortical thickness typically involve region-based analyses, which aggregates values within gross anatomical regions. In line with prior literature (Sun et al., 2023; van Erp et al., 2018), our regional analyses did reveal cortical thinning in individuals with psychosis compared to controls. However, the VCDM approach demonstrated substantially larger effect sizes, suggesting superior sensitivity to disease-related cortical abnormalities. For context, the ENIGMA schizophrenia study (van Erp et al., 2018) used conventional region-based FreeSurfer analyses and reported widespread cortical thinning across nearly all Desikan–Killiany atlas regions, with regional effect sizes ranging from –0.54 (right fusiform gyrus) to –0.08 (left pericalcarine cortex) and global hemispheric effects of d = –0.53 (left) and –0.52 (right). A voxel-based morphometry (VBM) meta-analysis of antipsychotic-naïve psychosis reported moderate-to-large gray matter reductions (Hedges’ g ≈ 0.69 in clinical high-risk and 0.83 in first-episode psychosis; (Fusar-Poli et al., 2012)). By contrast, the present VCDM analyses yielded substantially larger effects (d > 1), underscoring its potential advantage in detecting robust cortical abnormalities. The approach assesses cortical thickness in a vertex-wise manner and compares individual data points to a large, normative reference population. This normative mapping allows for deviations to be expressed relative to what would be expected for age and sex, rendering results more interpretable in both research and clinical contexts.

Using VCDM, we found that individuals with psychosis had significantly more vertices showing cortical thinning compared to controls, while the pattern of cortical thickening was similar across groups. These findings were not only more widespread but also associated with greater effect sizes than those derived from traditional region-based analyses. Cortical thinning in schizophrenia has been widely documented, particularly in frontal and temporal regions, with greater reductions observed in chronic cases and those with poorer outcomes (Godwin et al., 2018; Sun et al., 2023; van Erp et al., 2018). To evaluate whether regional focus could improve sensitivity, we examined group differences in the frontal and temporal lobes—areas most commonly implicated in schizophrenia. However, restricting analyses to these regions resulted in effect sizes that were smaller than those derived from whole-brain VCDM analysis. This suggests that structural abnormalities in schizophrenia are not localized but instead involve distributed cortical networks.

Among individuals with psychosis, greater cortical thinning was associated with increased positive symptom severity (measured via PANSS) and, in a separate dataset, with more severe negative symptoms (measured via SANS or CAINS). This aligns with prior research linking cortical structural changes with core symptom domains of schizophrenia (Walton et al., 2017; Walton et al., 2018; Y. Xiao et al., 2015a, 2015b). Interestingly, we also observed that a higher percentage of abnormally thick cortices was positively associated with working memory performance in the psychosis group. This dichotomy in behavioral relationships to cortical thinning and thickening, may explain prior research which suggests that cognitive impairments and psychosis are distinct and dissociable processes in schizophrenia (Dominguez Mde et al., 2009; Hanford et al., 2019; Heinrichs et al., 2015). Therefore, it has been proposed that cognitive deficits be distinguished from illness-specific brain anomalies, which could serve as potential targets for treatment (Hanford et al., 2019). In healthy controls, by contrast, cortical thickness was inversely related to vocabulary and executive functioning scores, though this effect was attenuated or reversed when using age-adjusted cognitive scores. This is consistent with a confounding effect of normal age-related developmental trajectories involving gradual cortical thinning beginning in early childhood (Bethlehem et al., 2022). The significance of the observed correlation between cortical thickness and emotion recognition in controls is unclear but may reflect autism spectrum traits within the cohort, which are often linked to increased cortical thickness due to atypical neurodevelopmental processes such as reduced synaptic pruning and delayed maturation (Khundrakpam et al., 2017). In our smaller BRAINS cohort, we lacked cognitive data but found associations between cortical thickness deviations and disorganization, which have been linked to all domains of neurocognition (Ventura et al., 2010; Vignapiano et al., 2019). As both disorganization and cognitive impairment are strongly associated with functional disability and poor prognosis (Rocca et al., 2018), these findings further support assessment of cortical thickness—particularly when measured via VCDM—as a potential prognostic marker.

Our heritability analyses indicated high genetic influence on our cortical thickness metrics, consistent with previous findings in the literature (Kruggel & Solodkin, 2020; Panizzon et al., 2009). This supports the robustness and biological validity of the VCDM approach and suggests that cortical thickness deviations may be valuable in understanding genetic liability and disease risk. However, as with most psychiatric phenotypes, environmental factors likely play a modifying role, offering a potential avenue for intervention.

VCDM holds promise as a clinician-friendly, developmentally anchored method that provides individualized assessments of brain structure relative to normative expectations. Its potential application in early detection—particularly during the prodromal phase of psychosis—is notable. Furthermore, it may offer enhanced sensitivity for monitoring treatment effects and illness progression, potentially guiding more timely therapeutic adjustments.

Nonetheless, several limitations should be acknowledged. Firstly, the VCDM approach relies on selecting an appropriate reference population for comparison. Our current study used a large young adult sample primarily from the St. Louis, Missouri, which may limit generalizability. To account for age-related cortical changes and improve applicability, future studies should adjust references by age group and consider a more globally representative sample. Secondly, antipsychotic medication, particularly typical antipsychotics, have been associated with cortical thinning (Tuominen et al., 2025; van Haren et al., 2011; Voineskos et al., 2020), which may confound group differences. Future studies focusing on antipsychotic-naïve individuals—such as clinical high-risk (CHR) cohorts who later convert to psychosis—could help clarify medication-independent structural abnormalities. Thirdly, our study also did not account for substance use, which occur relatively frequently in psychotic populations (Khokhar et al., 2018; Nesvag et al., 2015) and can also influence cortical development (Lorenzetti et al., 2016; Panizzon et al., 2009; P. Xiao et al., 2015a, 2015b), although it has been argued that the compounding effect of substance use on gray matter deficits in schizophrenia may be minimal (Quinn et al., 2018). Fourthly, scanner-related effects must also be considered. Our data were collected exclusively on Siemens 3 T scanners with optimized T1-weighted imaging parameters. While this enhances internal consistency, generalizability to other scanner types and field strengths must be validated. Given the high similarity of cortical thickness measures and pTHIN/pTHICK distributions across datasets, additional scanner harmonization procedures (e.g. ComBat) were not applied, as they were unlikely to materially affect the observed results. Finally, as an extent threshold has not yet been formally established for the VCDM approach, we displayed all cluster sizes in the cortical surface renderings; however, smaller clusters should be interpreted with caution, as they may reflect noise rather than robust anatomical effects. Future research should evaluate the robustness of VCDM across diverse imaging platforms and acquisition protocols.

In summary, VCDM offers a novel, sensitive, and developmentally anchored approach to assessing cortical thickness that outperforms traditional regional methods in detecting brain abnormalities associated with psychosis. Its individualized, normative-based outputs have the potential for clinical utility in diagnosis, prognosis, and treatment monitoring. Continued research is warranted to examine its utility in early detection, across development, and in response to treatment interventions—especially in diverse and medication-naïve populations.

## Data Availability

Data will be available upon request to the corresponding author.
